# Clinical, cognitive, and morphometric profiles of progressive supranuclear palsy phenotypes

**DOI:** 10.1007/s00702-023-02591-z

**Published:** 2023-01-26

**Authors:** Marta Campagnolo, Luca Weis, Carmelo Fogliano, Valeria Cianci, Michela Garon, Eleonora Fiorenzato, Miryam Carecchio, Florinda Ferreri, Patrizia Bisiacchi, Angelo Antonini, Roberta Biundo

**Affiliations:** 1 Parkinson’s Disease and Movement Disorders Unit, Department of Neuroscience, Centre for Rare Neurological Diseases (ERN-RND), University of Padova, Padova, Italy; 2 Department of General Psychology, University of Padova, Padova, Italy; 3 Study Center for Neurodegeneration (CESNE), University of Padova, Padova, Italy; 4 Department of Neuroscience, University of Padova, Padova, Italy; 5 IRCCS San Camillo Hospital, Venezia, Italy

**Keywords:** Progressive supranuclear palsy PSP, Clinical phenotype, MRI, Cognitive dysfunction, Functional disability

## Abstract

The International Parkinson’s and Movement Disorder Society (MDS) criteria for progressive supranuclear palsy (PSP) have broadened the clinical spectrum of the disease and established phenotypic characterization according to the predominant manifestation at onset. The objective of this study is to describe clinical/cognitive and imaging features of a monocentric cohort of PSP patients, highlighting different patterns of functional disability according to the assigned phenotype. We retrospectively reviewed clinical/imaging data of 53 PSP patients diagnosed with probable PSP according to the MDS criteria and 40 age/sex-matched healthy controls (HCs). Neurological/neuropsychological assessments were performed using standardized scales, as well as comprehensive magnetic resonance imaging (MRI) morphometric measurements. In our cohort, there were 24/53 PSP-RS (Richardson’s syndrome), 13/53 PSP-P (Parkinsonism), 7/53 PSP-PGF (Progressive gait freezing), and 9/53 PSP-Cog (Cognitive impairment). PSP-Cog presented the worst motor profiles, the highest percentages of dementia and impaired functional autonomy; 4/9 PSP-Cog and 2/7 PSP-PGF died. PSP-P had the lowest motor/cognitive burden. All MRI parameters had good discriminative efficacy vs. HCs, with P/M 2.0 discriminating PSP-PGF from PSP-RS and PSP-Cog. We highlighted discrete clinical and imaging patterns that best characterize different PSP phenotypes. PSP-Cog and PSP-PGF/RS manifest greater incidence of dementia and motor disability, respectively, while PSP-P has a more benign course. The identification of different phenotypes may be the expression of different progression patterns requiring tailored approaches in terms of follow-up and treatment. These findings support the concept of discrete patterns of Tau pathology within the PSP spectrum and encourage research for phenotype-specific outcome measures.

**Supplementary Information:**

The online version contains supplementary material available at 10.1007/s00702-023-02591-z.

## Introduction

Progressive supranuclear palsy (PSP) is a rare adult-onset neurodegenerative disorder characterized by 4-repeat tau pathology in cortical and subcortical brain regions. The initial NINDS-SPSP (National Institute of Neurological Disorders and Stroke and Society) criteria recognized only one phenotype, described by Richardson, Steele and Olszewski in 1964 (Richardson’s syndrome, PSP-RS), characterized by vertical gaze palsy, postural instability, falls (Richardson et al. 1963; Litvan et al. 1996). However, a significant proportion of cases did not manifest typical PSP-RS features at onset, or they became apparent after several years, making the diagnosis complex. The interest in the definition of phenotypes other than PSP-RS (Williams et al. 2005; Hoglinger et al. 2017) led to the development of the 2017 International Parkinson’s and Movement Disorder Society (MDS) PSP criteria, with the aim to increase diagnostic specificity and sensitivity (Ali et al. 2019). The updated criteria have determined the recognition of new entities and classify patients according to the predominant manifestation at onset: Parkinsonism (PSP-P), progressive gait freezing (PSP-PGF), frontal presentation (PSP-F), ocular motor dysfunction (PSP-OM), speech/language disorder (PSPSL), and cortico-basal syndrome (PSP-CBS).

However, these criteria have some limitations. In particular, different attributes belonging to discrete levels of certainty (according to the 2017 criteria) and with no reference to the severity of the symptoms may be simultaneously observed. Therefore, the definition of a specific phenotype may be challenging (Picillo et al. 2019; Grimm et al. 2019). This issue applies particularly to patients with early cognitive alterations, currently defined by three discrete level features (speech disorder, frontal/behavioral presentation, and cortico-basal syndrome) often overlapping (Picillo et al. 2019; Grimm et al. 2019; Rohrer et al. 2010; Burrell et al. 2014; Fiorenzato et al. 2019). Similarly, in patients with clinical manifestations included in the akinesia core (progressive gait freezing, parkinsonism), discrete attributes may be simultaneously present.

Moreover, to date, several clinical/cognitive and neuroimaging studies have focused on optimizing PSP diagnostic accuracy including recently published magnetic resonance imaging (MRI) morphometric indices which have been proposed for clinical trials (Morelli et al. 2011; Boxer et al. 2017; Picillo et al. 2020; Quattrone et al. 2018, 2021). However, their relevance in discriminating PSP phenotypes needs more evidence.

Finally, an increased body of literature supports that non-motor and motor features both impact on patients’ disability. However, the current PSP criteria are focused mostly on the motor aspects with the purpose of differentiating the clinical phenotype based on the presence of different patterns of prevalent symptoms.

Hence, the aims of our retrospective cross-sectional study were to investigate (i) the applicability/feasibility of the most recent published PSP criteria in detecting PSP phenotypes in a monocentric Italian cohort, (ii) to highlight the different pattern of disability as result of multidimensional symptoms (motor, cognitive, and behavioral alterations) and not only of the predominant characteristics defining each PSP phenotype, and (iii) to test whether different MRI indices and measures would distinguish patients according to clinical classification and would discriminate them from HC.

## Methods

### Patients’ recruitment

From a total of 2764 patients evaluated at the Parkinson’s Disease and Movement Disorders Unit in Padova (January 2016–2021), we identified 66 subjects diagnosed with probable PSP according to the 2017 Movement Disorder criteria (Hoglinger et al. 2017). To have a homogeneous cohort, we considered only 53 patients who had a complete clinical (motor, neuropsychological, and behavioral) assessment, a detailed recollection of symptoms reported at onset, and in 47/53 MRI scanning. DAT-Scan SPECT was also available to confirm loss of dopamine nerve terminals. These patients had a relatively similar range of disease duration of 3–8 year from diagnosis of Parkinsonism with a median of 5 years. To assess survival, we also recorded if any of these 53 patients subsequently died.

PSP patients were compared with 40 age- and sex-matched healthy controls (HCs) recruited as part of the NHS ongoing project “Validation of Mild Cognitive Impairment criteria in Italian Parkinson’s disease patients” (GR-2016-02361986).

### PSP phenotype characterization

A multidisciplinary team of at least two movement disorders’ specialists, including neurologists, neuropsychologists, and biotechnologists, independently reviewed all data collected for each subject and retrospectively categorized our cohort according to the onset symptoms following the 2017 Movement Disorder criteria (Hoglinger et al. 2017). However, with the exception of PSP-RS, the allocation to specific PSP phenotypes was in some cases difficult due to the concomitant report of clinical features belonging to domains where attributes, being simultaneously present, could not be discretely allocated, namely the cognitive (speech disorder, frontal/behavioral presentation, and cortico-basal syndrome) and the akinesia domain (progressive gait freezing, parkinsonism). Therefore, we decided to apply the Multiple Allocations eXtinction (MAX) rules of temporal order of predominance types for patients’ allocation (Grimm et al. 2019). Given the small number of cases, we preferred to include all patients with predominant cognitive features in one phenotypic class which we defined PSP-Cog. Eventually, four phenotypes were identified: PSP-Cog, PSP-PGF, PSP-P, and PSP-RS.

### Clinical assessment

A complete neurological examination was performed. Disease severity was assessed using the PSP-Rating Scale (PSPRS) and the Movement Disorder Society Unified Parkinson’s Disease Rating Scale (MDS-UPDRS) (Golbe and Ohman-Strickland 2007; Antonini et al. 2013) part III (motor examination). HCs had no history of neurologic, psychiatric, or other major medical illnesses. Exclusion criteria for patients and HCs were history of neuroleptic use within the past 6 months, evidence of vascular lesions (lacunar infarctions in the basal ganglia and/or subcortical vascular lesions with diffuse periventricular signal alterations), or normal pressure hydrocephalus as assessed with FLAIR-3d MRI sequence. Study participants gave written informed consent. The study was approved by the Local Institutional Ethical Committee, according to the Helsinki Declaration.

### Neuropsychological evaluation

All patients underwent an extensive neuropsychological evaluation consisting of a cognitive assessment with at least two tests for each of the five cognitive domain (i.e., attention/working memory, executive, memory, language, and visuospatial/visuo-perceptive functions) and a behavioural screening, according to a previously described protocol (Fiorenzato et al. 2019). Mini-Mental State Examination (MMSE) and Montreal Cognitive Assessment (MoCA) scales (Fiorenzato et al. 2019) were used to assess the global cognitive functioning. Autonomy in daily life was assessed using activities of daily living (ADL) (Katz and Akpom 1976) and instrumental ADL (IADL) (Graf 2008) scales. Applying instruments that clearly separate the functional consequences of motor and cognitive disabilities is critical in movement disorders. Considering the lack of validated tools in PSP we employed the Parkinson’s Disease-Cognitive Functional Rating Scale (PD-CFRS) (Kulisevsky et al. 2013) to investigate the impact of cognitive alterations on patient’s activities minimizing possible biases derived from motor impairment. Depression was evaluated with the Beck Depression Inventory-II (DBI-II) (cut-off = 14), apathy with the Starkstein Apathy Scale (AS) (cutoff = 14), impulsivity with the Barratt Impulsiveness Scale (BIS) (cut-off = 60), anxiety with the StateTrait Anxiety Inventory (STAI-I, STAI-II) (cut-off = 40). Scores above the respective cut-offs were suggestive of clinically significant symptoms.

Well-being and quality of life were evaluated using the Parkinson’s Disease Questionnaire (PDQ-8) (Jenkinson and Layte 1997). The Neuropsychiatric Inventory-Questionnaire (NPI-Q) (Cummings 1997) was administered to caregivers, to assess the presence and severity of patient’s neuropsychiatric symptoms.

### Cognitive status

Due to the lack of PSP specific criteria for assessing cognitive statuses, dementia and mild cognitive impairment (MCI) were diagnosed according to the published MDS Level II PD criteria (Litvan et al. 2012; Dubois et al. 2007). Namely, we classified patients as MCI, if the z score for a given test was at least 1.5 standard deviation (SD) below the appropriate norms in two tests (e.g., within a single cognitive domain or at least one test in two or more cognitive domains) in the context of intact autonomy in basic functional living (ADL) (Litvan et al. 2012). Probable dementia was diagnosed by experienced neuropsychologists and based on performance at extensive neuropsychological tests (impairment in more than one cognitive domain), evaluation of functional autonomy (deficits severe enough to impair daily life), as well as clinical interview (behavioral or cognitive caregiver’s report; presence of behavioral symptoms).

### Cross-sectional negative variables

Lack of functional autonomy (ADL < 6&PD-CFRS > 6), presence of dementia, and severe motor disability [Hoehn and Yahr (H&Y) > 3] were evaluated for each phenotype and considered as quality of life and wellbeing’s modulator factors.

### MRI protocol

Forty-seven out of 53 probable PSP patients (47/53, 88.7%) and all 40 HCs underwent a comprehensive standardized MRI clinical protocol including a 0.9 mm isotropic T1-weighted 3D, 1 mm isotropic T2-weighted 3D or 1 mm isotropic T2-weighted 2D, and a 1 mm isotropic FLAIR 3D, simultaneously to the neuropsychological assessment. In 19 PSP patients (36%) and 29 HC (73%), MRI images were acquired on a Philips Ingenia 3 T scanner with 32-channel head coil. The remaining subjects were acquired using a Philips Achieva 1.5 T scanner with an 8-channel head coil. All MRI images were collected within 1 week from clinical assessment.

### Morphometric measurements

Midbrain-based measures were calculated, including mid-sagittal pons (P)-midbrain (M) areas, middle cerebellar peduncles to superior cerebellar peduncles ratio (MCP/SCP), P/M (pons area to midbrain area ratio), MRPI (Magnetic Resonance Parkinsonism Index), as well as P/M 2.0 and MRPI 2.0 (Morelli et al. 2011; Whitwell et al. 2017). All measures were manually assessed by drawing ROIs on the anatomical T1-weighted 3D. To minimize the inter-participant variability due to head position within the head coil, all the anatomical T1 were (1) previously manually corrected for realignment using the anterior–posterior commissure and the mid-sagittal point as landmark to the standard MNI template, and (2) reconstructed the 0.9 mm isotropic axial, sagittal, and coronal orthogonal views. Each measure was computed according to published methods by the same biotechnologist (LW) with more than 10 year experience in neurodegenerative diseases and blinded to diagnosis and phenotypic attribution.

### Statistical analysis

Differences in characteristics (clinical, motor, cognitive, and morphometric) between PSP subgroups were assessed with Kruskal–Wallis or Chi-squared test followed by pairwise Mann–Whitney *U* test or Fisher’s exact test as appropriate.

Reference intervals (median + CI 95%) of morphometric indices for HCs and PSP subgroups were calculated using the robust method described in the Clinical and Laboratory Standards Institute (CLSI) Guidelines C28-A3 (Wayne 2008). The double-sided confidence intervals were estimated with the bootstrap method using 10,000 replications. Presence of outlier was checked with Reed et al.’s method (Reed et al. 1971). Values’ distributions of morphometric indices within PSP subtype and between HCs were compared using a non-parametric Kruskal–Wallis ANOVA using Mann–Whitney test for post hoc statistical significance assessment.

A receiver-operating characteristic (ROC) analysis was performed to evaluate the discriminant power of morphometric indices (P/M, MRPI, P/M 2.0 and MRPI 2.0) for each PSP subgroup vs. healthy controls. Due to the small sample size, PSP subgroup comparison analyses could not be run and only PSP-RS vs the other PSP subgroups together was performed. Indices with an AUC within range of 0.7–0.9 have a moderate accuracy, whereas AUC > 0.9 have a high accuracy. A Forest plot was used to compare the indices’ AUC distributions among PSP subtypes.

Effect of MRI scanner (1.5 T vs. 3 T) on PSP and HC morphometric measure distribution was evaluated with a two-way ANOVA including morphometric measures as dependent variable, diagnosis, and scanner as factors. It was evaluated if there was an effect on dependent indices. Moreover, the interaction scanner × diagnosis was evaluated to assess if scanner dissimilarities could have an effect in testing differences among PSP subtypes, especially the within PSPs’ discriminative accuracy.

Pearson’s Chi-square statistic was used to compare the presence of each neuropsychiatric symptoms, and the frequency of distressful features (ADL < 6&PD-CFRS > 6, H&Y > 3, dementia) among PSP subtypes. We calculated for each PSP phenotype the frequencies of neuropsychiatric symptoms as assessed by NPI-Q without any statistical comparison analysis between PSPs, due to the small sample size. Statistical analyses were run using IBM-SPSS 25 (IBM SPSS Inc., Chicago, Illinois, United States) and significance threshold was set at *p* ≤ 0.05.

## Results

### Demographic and clinical characterization

According to the 2017 PSP Movement Disorder criteria, among the 53 patients fulfilling the criteria for probable PSP, 24 (45.3%) were defined as PSP-RS, 13 (24.5%) as PSP-P, 9 (17%) as PSP-Cog, and 7 (13.2%) as PSP-PGF. PSP-Cog simultaneously presented multiple cortical cognitive symptoms including apraxia, speech and language disorders, and frontal features that are listed in the proposed domain attributes.

No statistically significant differences were observed among clinical phenotypes regarding age of onset, education, and disease duration.

Scores for the UPDRS part III were higher in PSP-Cog and in PSP-PGF compared to PSP-P and PSP-RS (*p* = 0.003 and *p* = 0.003 respectively).

The PSPRS total score did not differ among the four phenotypes, despite a trend for a higher total score in PSP-Cog. Subitems analyses showed higher scores in the limb motor items (Katz and Akpom 1976; Graf 2008; Kulisevsky et al. 2013; Jenkinson and Layte 1997; Cummings 1997; Litvan et al. 2012) in PSP-Cog compared to PSP-RS and PSP-P (*p* = 0.01), and in mentation items (Rohrer et al. 2010; Burrell et al. 2014; Fiorenzato et al. 2019; Morelli et al. 2011) in PSP-Cog, PSP-RS, and PSP-PGF compared to PSP-P (*p* = 0.008).

There was a strong trend for greater proportion of H&Y stage ≥ 3 in PSP-RS patients (*p* = 0.051).

### Functional and cognitive features

A strong trend for worse ADL scores was observed in PSP-Cog compared to PSP-P (*p* = 0.054). IADL scores resulted lower (more impaired) in PSP-Cog compared to PSP-RS (*p* = 0.03). In line with ADL and IADL scores, PSP-Cog showed the highest PD-CFRS score compared to PSP-P and PSP-PGF (*p* = 0.012).

Median MMSE corrected scores were the lowest in PSP-Cog compared to the other groups, although seen only as a strong trend (*p* = 0.054). PSP-Cog also presented the lowest MoCA corrected scores vs. all other phenotypes (*p* = 0.008).

A higher prevalence of dementia was observed in PSP-Cog (33.3%), although no statistically significant difference was observed when compared to other subgroups. Of note, in PSP-P and PSP-PGF groups, none of the patients were demented. Level II MCI status was diagnosed across the four phenotypes, with high prevalence in patients with PSP-RS and PSP-PGF (70.8% and 83.3%, respectively).

No differences across phenotypes were observed in quality of life (PDQ-8).

See Table 1 for a detailed description of each phenotype at time of assessment.

**Table 1:** Demographical, clinical, and motor phenotypes characteristic

	PSP-Cog (*N* = 9)		PSP-P (*N* = 13)		PSP-PGF (*N* = 7)		PSP-RS (*N* = 24)		Kruskal–Wallis ANOVA	Cog vs. RS	Cog vs. P	PGF vs. P	PGF vs. RS	RS vs. P	Cog vs. PGF
	Median	IRQ 2.5–97.5	Median	IRQ 2.5–97.5	Median	IRQ 2.5–97.5	Median	IRQ 2.5–97.5	*p* value						
Age (years)	71	64–79	71	58–74	75	62–84	71	55.3–84							
Education (years)	9.0	5–22	13	5–19	8	5–23	8	4–18							
Sex (M %)	44.4%		53.8%		50.0%		33.3%		0.5443 0.1003 0.6451 0.6905						
Age at symptoms’ onset (years)	69	61–71	66	54–71	67	57–78	67	52–81							
Disease duration (years.)	4	3–8	4	3–7	5	3–8	4	3–8	0.3858						
MDS-UPDRS															
Part III	54	36–68	29	9–48	58	38–61	42	17–77	**0.0029**	*	*	*		*	
H&Y ≥ 3	55.6%		53.8%		66.7%		91.3%		0.0514						
PSP-rating Scale															
Bulbar	2	0–4	2.5	1–6	1.5	1–2	3.5	1–7	0.2985						
Gait and midline	10	5–20	7	4–15	15	14–16	10.5	3–17	0.1363						
History	8	5–16	4.5	3–13	6	5–7	8.5	4–19	0.1811						
Limb motor	7	6–12	5.5	2–8	6	5–7	4.5	2–6	**0.0141**	*	*				
Mentation	6	0–11	0	0–1	1	0–2	3.5	1–6	**0.0086**		*	*		*	
Ocular motor	9	6–13	7.5	3–12	5.5	2–9	8	2–14	0.5342						
Total score	42	30–66	27.5	15–49	35	35–35	40	21–56	0.0963						
PDQ-8	10	0–16	9	2–22	8	4–9	11	4–24	0.3221						
IADL	2	0–8	5	2–8	5	1–8	4	1–8	**0.039**	*					
ADL	2	0–6	5	2–6	3.5	2–6	4	1–6	0.054		*				
PDCFRS	17	1–21	3	0–7	2	1–19	7	3–13	**0.012**		*		*	*	*
MoCA (corr. score)	14.98	7–25.0	20.7	18.7–27.7	21.4	15.9–24.1	19.9	13.4–28.4	**0.008**	*	*				*
MMSE (corr. score)	21	7–30	26.3	13.7–30.0	25	19.7–27.7	25.3	11.55–30.0	0.054						
CNT/MCI/dementia (%)	11.1/55.6/33.3		38.5/61.5/0		16.7/83.3/0		12.5/70.8/16.7		0.1575						

PSP phenotypes were defined based on predominant symptoms at onset. In particular, PSP-Cog included patients with predominant cognitive manifestation at onset

Bold values indicate statistically significant *p* values

*MDS-UPDRS* MDS-Unified Parkinson’s Disease Rating Scale, *PDQ-8* Parkinson’s Disease Quality of Life Questionnaire 8-item scale, *IADL* Instrumental Activities of Daily Living scale, *ADL* Activities of Daily Living scale, *PDCFRS* Parkinson’s disease cognitive functional rating scale, *MoCA* Montreal Cognitive Assessment scale, *MMSE* Mini-Mental State Examination, *CNT* preserved cognitive status, *MCI* PD with mild cognitive decline

### Behavioral features

**Fig. 1:**
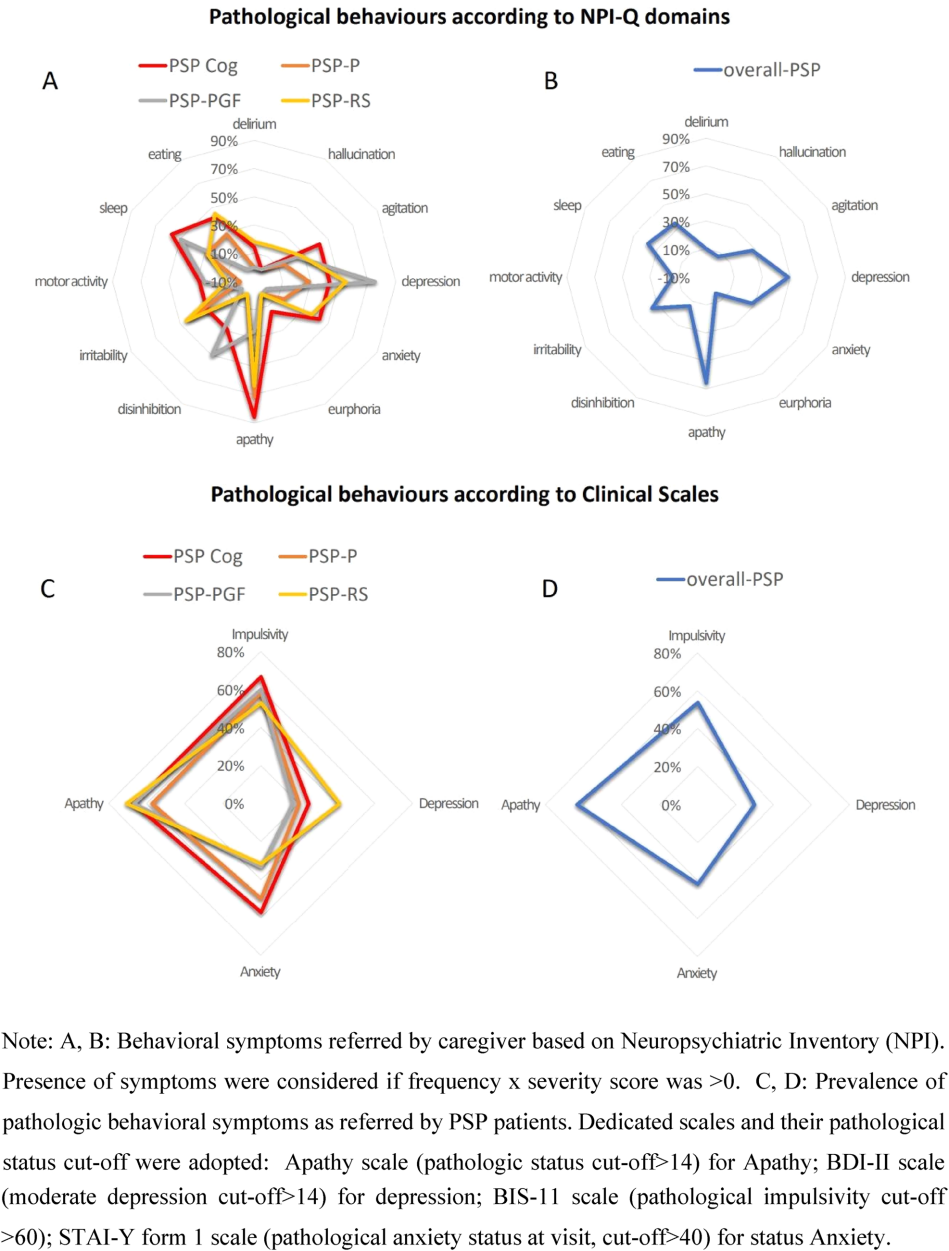
Neuropsychiatric symptoms across PSP phenotypes

From the Neuropsychiatric Inventory-Questionnaire (NPI-Q), caregivers reported that apathy was the most common neuropsychiatric symptom across each phenotype except PSP-PGF (85.7% of PSP-Cog, 71.4% of PSP-P, and 63.6% of PSP-RS), followed by depression (75% of PSP-PGF, 54.4% of PSPRS, and 42.9% of PSP-Cog) except PSP-P (25%). Sleep disturbances were quite common among PSP patients, particularly in PSP-Cog and PSP-PGF (57% and 50%, respectively, Fig. 1A).

Patients-reported frequency of significant abnormal behaviors (by means of published cut-offs) confirms a substantial overlap between PSP phenotypes, with apathy described as the most common symptom (in 66.7% of PSP-Cog, 57.1% of PSP-P, 66.7% of PSP-PGF, 70.6% of PSP-RS, and overall prevalence of 63.3%) followed by impulsivity (in 66.7% of PSP-Cog, 58.3% of PSP-P, 60% of PSP-PGF, 52.9% of PSP-RS, and overall prevalence of 53.6%), state anxiety (overall prevalence 41.7%, mostly in PSP-Cog and PSP-P [57.1% and 50%]) and trait anxiety (overall prevalence 51.4%, mostly in PSP-PGF and PSP-RS patients [50% and 47.4%]). Reported depression was less frequent (overall prevalence 29.7%, mostly in PSP-RS [41.2%]) (Fig. 1B).

### MRI morphometric indices

MRPI, P/M ratio, MRPI 2.0, and P/M 2.0 were calculated for all patients and compared to normative values. Forty HCs were considered to determine normative values according to the robust method described in the CLSI Guidelines C28-A3 (Wayne 2008).

All four MRI indices were statistically significant different in PSP vs HC (overall non-parametric Kruskal–Wallis ANOVA *p* < 0.00001, post hoc Mann–Whitney *p* < 0.001).

**Fig. 2:**
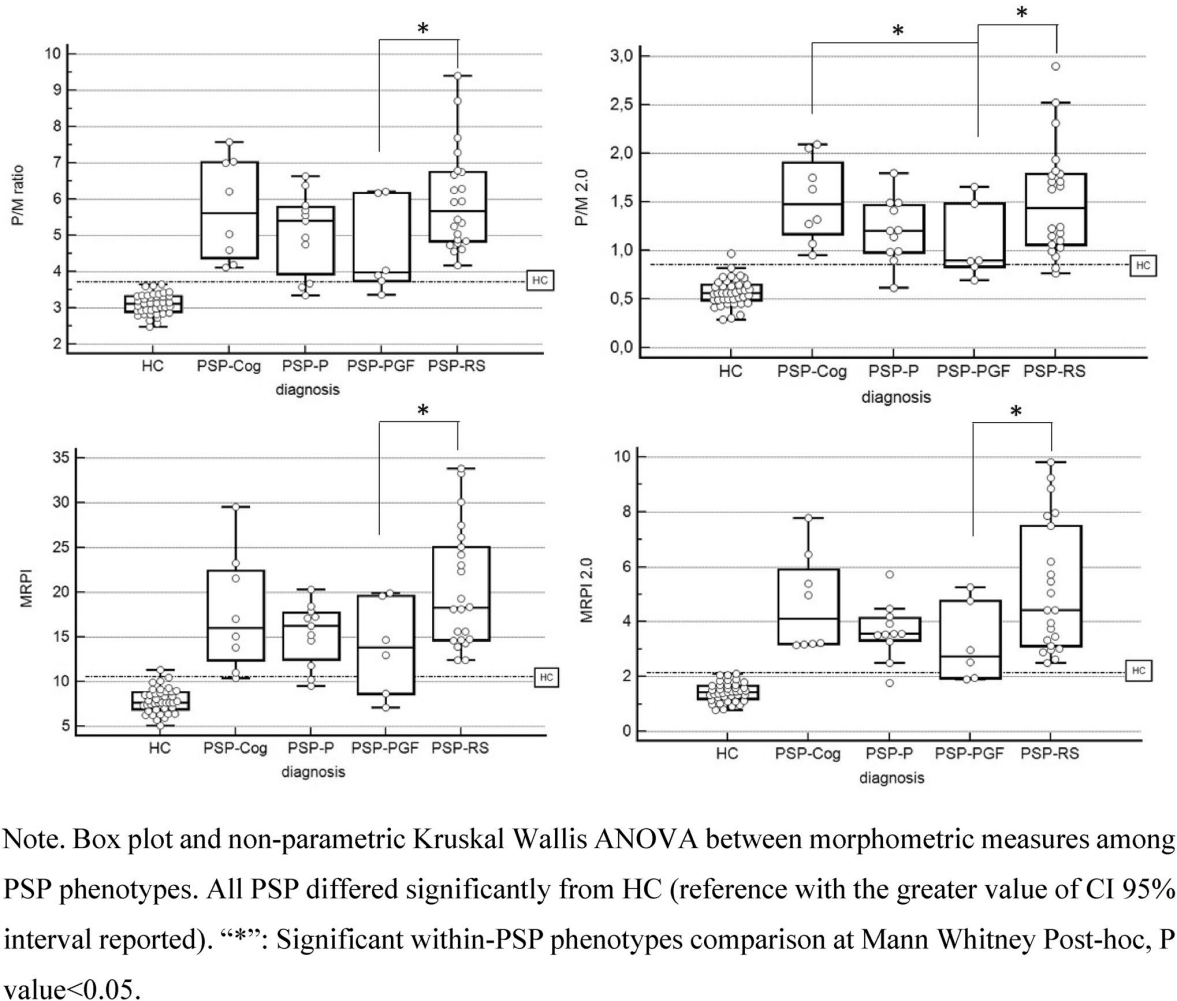
Comparison of morphometric measures among PSP phenotypes

Of note, P/M 2.0 showed significant sensitivity in discriminating PSP-PGF from PSP-RS and PSP-Cog (*p* < 0.05) (Fig. 2).

Overall, all morphometric indices accurately discriminated PSP from healthy controls (AUC_P/M ratio_ = 0.990, AUC_MRPI_ = 0,973, AUC_P/M2.0_ = 0.983, and AUC_MRPI 2.0_ = 0,993).

**Fig. 3:**
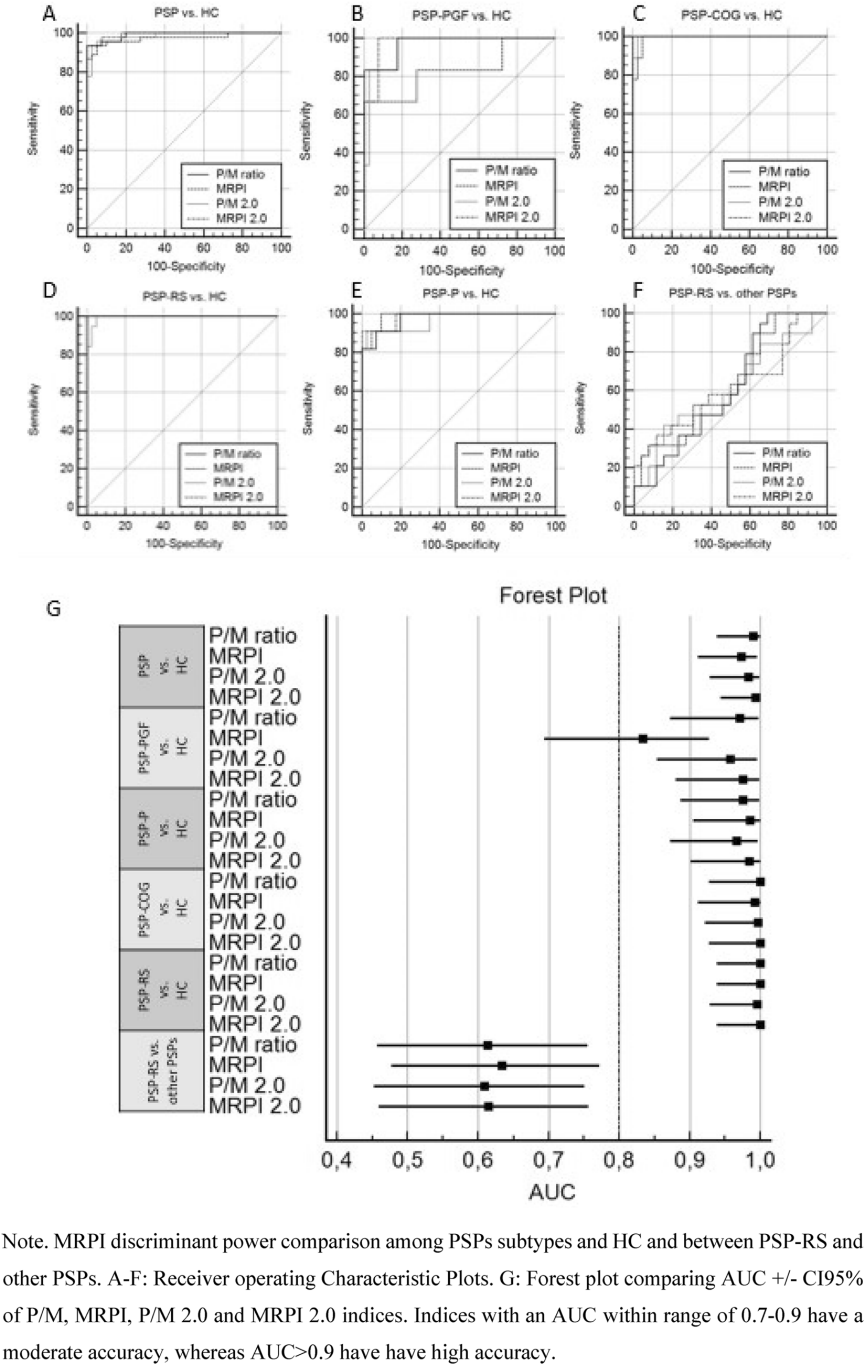
MRPI indices discriminative power of PSPs phenotypes vs. normal population

Regarding PSP subgroups vs. HC, all indices performed similarly with high discriminative accuracy (AUC > 0.9) except MRPI in PSP-PGF. When adopted within PSP subtypes, none of the indices have reached significance in discriminating PSP-RS vs. other PSPs (AUC_P/M ratio_ = 0.613, AUC_MRPI_ = 0.634, AUC_P/M2.0_ = 0.609, AUC_MRPI 2.0_ = 0.615) (Fig. 3).

Finally, two ANOVA results on manually measures confirm a global effect of diagnosis on all the morphometric indices (*p* < 0.001). Global effect of scanner was not significant in each index (scannerP/M ratio *p* = 0.675, scanner _MRPI_ *p* = 0.897, scanner _P/M2.0_ *p* = 0.356, scanner _MRPI 2.0_ *p* = 0.526). The interaction between scanner and diagnosis is significant in MRPI only (scanner*diagnosis _MRPI_ *p* = 0.039), while for the other indices, there was no impact of scanner on the discriminant power (scanner*diagnosis _P/M ratio_ *p* = 0.887, scanner _P/M2.0_ *p* = 0.871, scanner _MRPI 2.0_ *p* = 0.114) (e-Fig. 2).

Data regarding MRI morphometric indices and normative values are reported in the Supplementary Materials.

### Negative cross-sectional variables across PSP phenotypes

**Fig. 4:**
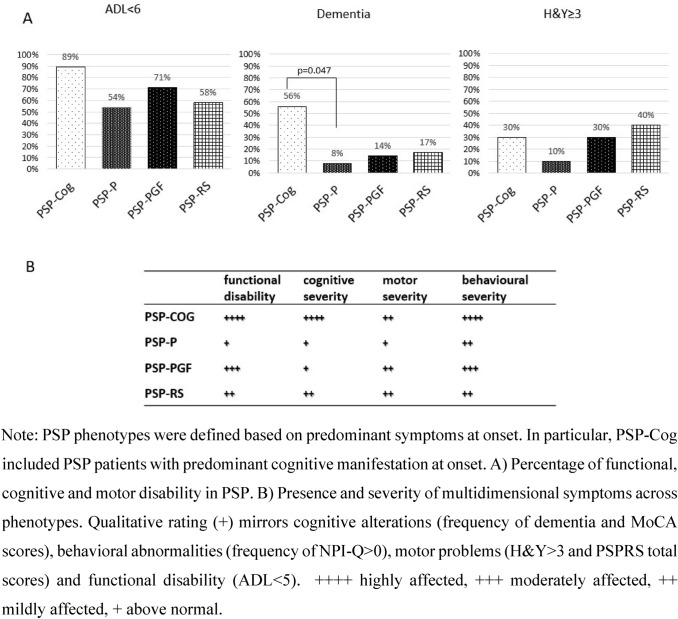
Disease characterization across PSP phenotypes

PSP-Cog showed higher percentages of dementia (only seen significantly different when compared to PSP-P) and impaired functional autonomy (ADL < 6&PD-CFRS > 6), although they were present across each phenotype. PSP-RS showed the highest percentage of H&Y scores ≥ 3 although only observed as a trend (Fig. 4). Overall, PSP-Cog presented the most altered functional independence together with the worst motor profile (higher UPDRS part III and limb motor subitems of the PSPRS scores) but lower percentages of patients with H&Y ≥ 3. In PSP-PGF, the motor impairment including balance plays a crucial role in limiting autonomy, whereas no major cognitive deficits were detected. However, high rates of depression were disclosed by specific questionnaires. See Fig. 4(A) to identify the disability pattern in the context of each PSP phenotype, defined according to the current diagnostic criteria, with focus on copresence of multidimensional symptoms (B).

From the Hospital database, 9/53 patients in our cohort (4/9 PSP-Cog and 2/7 PSP-PGF) died within 8 years from clinical diagnosis. None of the PSP-P died within 8 years (e-Fig. 3, Supplementary Material).

## Discussion

The first aim of our study was to retrospectively evaluate clinical, cognitive, and imaging features that best characterize a monocentric cohort of PSP patients diagnosed with discrete phenotypes classified according to the MDS diagnostic criteria. A correct diagnostic definition in the early stages of the disease still constitutes the main issue both in the clinical and research setting (Respondek and Höglinger 2016). We did not want to create new PSP subtypes, criticizing the current criteria, but to evaluate the a-priori defined phenotypes (based on the main symptoms) from a wider perspective, including the functional outcome. When in the presence of concomitant multiple symptoms, a proper subtype allocation might be challenging. Therefore, in our cohort, patients’ stratification was performed according to Multiple Allocations eXtinction (MAX) rules (Grimm et al. 2019) with a retrospective categorization based on the predominant clinical features in the earliest phase of the disease. We also found that application of criteria is easier in presence of motor symptoms, while it is more challenging when patients report discrete but widespread disturbances at onset as in case of PSP-Cog and PSP-PGF. Regarding cognitive impairment, in light of the small number of cases for each attribute and supported by recent evidence, we decided to merge the three subtypes (PSP-CBS, PSP-F, and PSP-SL) into one PSP-Cog phenotype, based on the previous studies also considering patients with PSP-CBS, PSP-F, and PSP-SL as a whole, defined as PSP-cortical group or identifying them as PSP cortical phenotypes (Jabbari et al. 2020; Guasp et al. 2021; Tsuboi et al. 2005; Kovacs et al. 2020).

With regard to our second aim, different patterns of disability were observed. PSP-Cog patients, who presented the worse functional independence together with the worst motor profile, showed higher scores in the UPDRS part III and limb motor subitems of the PSPRS (possibly due to the marked apraxia observed in the PSP-CBS patients). Interestingly, PSP-Cog presented smaller proportion of patients with H&Y ≥ 3, suggesting that severe balance problems were less common than in other phenotypes. This discrepancy, together with higher scores in the mentation items of the PSPRS, suggests that in PSP-Cog, cognitive deficits (mainly apraxia and comprehension problems) impact heavily on functional independence, possibly to a greater extent than gait and balance. By contrast, PSP-PGF patients showed a similar disability pattern as in PSP-Cog, despite the absence of major cognitive deficits (none had dementia), but in presence of high rates of depression. Indeed, PDCFRS score was in the normal range while ADL was reduced, suggesting that in this phenotype, the motor impairment including balance plays a crucial role in limiting autonomy. PSP-RS showed a mixture of cortical (presence of dementia) and subcortical (especially balance) disabilities as reflected by both ADL and PD-CFRS scores. Finally, PSP-P had a more benign features, with a lower motor and cognitive burden, and an overall lower degree of disability. Indeed, none of the PSP-P died within the observation period, in contrast with the other phenotypes. These findings support recent neuropathology data demonstrating differences in Tau burden brain distribution (higher cortical load in PSP-Cog, higher subcortical load in PSP-RS, and lowest in PSP-P) (Sánchez-Ruiz de Gordoa et al. 2022).

In line with the results on functional autonomy, in our cohort, the majority of deceased patients were PSP-Cog and PSP-PGF, suggesting that a thorough definition of clinical phenotype may help defining pattern of progression and eventually prognosis.

From a neuropsychological point of view, we found MoCA to be more sensitive than MMSE in differentiating PSP phenotypes. These results corroborate and expand previous findings (Fiorenzato et al. 2019, 2016), showing that MoCA is a sensitive tool to detect early cognitive dysfunctions in PSP-RS, as well as to discriminate cognitive performance within PSP phenotypes.

As observed in the previous studies (Picillo et al. 2019; Fiorenzato et al. 2019), dementia was common in our cohort. Among PSP-Cog and PSP-RS, respectively, 33% and 17% developed dementia within 3–8 years from onset. The current PSP-MDS criteria (Hoglinger et al. 2017) do not list dementia among the supporting features, although documenting its presence may be relevant to consider a possible diagnosis of PSP together with other suggestive core features as well as to tailor potential treatment strategies including palliative care.

Aligned with these considerations, the absence of dementia in our cohort was more suggestive of a PSP-P and PSP-PGF diagnosis, leading us to propose presence of cognitive decline may enhance diagnostic accuracy of specific phenotypes. Moreover, given the higher prevalence (> 50%) in PSP-PGF and PSP-RS, definition of specific MCI criteria in PSP is warranted. This should take into account the concomitant severe deficits common in PSP (e.g., oculomotor, dysarthria, akinesia, and dystonia) which can interfere with the cognitive workup.

An objective neuropsychological assessment seems to be critical in PSP patients, in light of the mismatch observed in our cohort between the objective assessment of MCI and subjective reports (PSPRS mentation item with self-reported cognitive deficits) or functional independence scores (PDCFRS > 3). These findings confirm that a clear-cut correspondence between subjective vs. objective cognitive deficits might be missing and that a formal assessment with comprehensive cognitive testing should be performed (Siciliano et al. 2021).

In line with the previous studies (Picillo et al. 2019; Painous et al. 2020; Meissner and Höglinger 2020; Moscovich et al. 2020; Belvisi et al. 2018), high prevalence of mood disorders (depression, apathy) and sleep disturbances was observed among non-motor symptoms. In our sample, alike previous evidence (Picillo et al. 2019), depressive symptoms were less prevalent than the apathetic symptoms. Overall, we did not find significant differences among phenotypes in terms of self-reported neuropsychiatric disturbances. Interestingly, in the NPI-Q caregiver report, higher levels of disinhibition and depression were described in PSP-PGF, while a lower prevalence of depressive and apathetic symptoms were reported in PSP-P and PSP-PGF, respectively. Noteworthy, caregivers overestimated the presence of depression, which was self-reported only by a small patients’ subgroup through the BDI-II scale. This discrepancy may be due to caregivers labelling apathetic symptoms as depression (Valentino et al. 2018). Notably, the presence of severe cognitive deficits can hamper patient’s self-evaluation, due to insufficient insight about behavioral deficits. Hence, we suggest considering both self-reported and caregiver’s assessment to obtain a more comprehensive evaluation of neuropsychiatric symptoms, and in turn ameliorate diagnostic accuracy.

Regarding neuroimaging findings, we recently contributed to numerous studies where several quantitative MRI parameters have been proposed as possible biomarkers. These include the Magnetic Resonance Parkinsonism Index (MRPI: pons area-midbrain area ratio × middle cerebellar peduncles width-superior cerebellar peduncles width ratio), the MRPI 2.0 (MRPI combined with 3rd ventricle width, a common finding in atypical Parkinsonisms, especially PSP), the pons area-to-midbrain area ratio (P/M), and the P/M 2.0 (P/M × 3rd ventricle width/frontal horns width). The measurement of the 3rdV width/internal skull diameter ratio (3rdV/ID) has also been validated in two independent cohorts, proving to be simple and reliable in identifying patients with PSP (Picillo et al. 2020; Quattrone et al. 2018, 2021, 2019). Despite their good sensitivity, these parameters require specific neuroradiological expertise that may not be available in all centres. In our cohort, in addition to significantly separate PSP from HCs (AUC > 0.983), P/M 2.0 provided preliminary evidence of sensitivity in discriminating among different PSP phenotypes (namely PSP-PGF from PSP-RS and PSP-Cog), although without significant accuracy (AUC_PSP-RS vs. other PSPs_ = 0.609) These findings suggest that MRI morphometric measures may selectively be considered as biomarkers, both in the early stages of the disease as supportive criteria. MRI morphometric measures showed a high variability between patients mainly related to the midbrain area which was in the normal range in many early PSP patients. In more advanced cases, manual measure of the width of left and right SCPs demonstrated worse reproducibility as the anatomical structure is very atrophic (< 2.5 mm). Finally, in line with literature, manual measure reliability was higher in state-of-the-art 3 T scanner due to the higher SNR assured by digital coil adopted as compared to the analogic ones adopted in the 1.5 T sample. Nonetheless, our results support that the discriminant power of the MPRI index is affected by the signal quality of the scanner used with better performances at 3 T, while the other indices (P/M, MRPI 2.0, P/M 2.0) performed similarly.

Further studies using the more reliable and recently validated automated morphometric measure of MRPI-2.0 applied on high-resolution T1-weighted anatomical acquisition (Quattrone et al. 2022) should address if this biomarker could accurately capture longitudinally the benefit of PSP treatment on disease progression in the context of possible clinical trials.

We acknowledge that our study has limitations, mainly the lack of neuropathological confirmation (even though patients were included with a high level of diagnostic certainty), the retrospective design and the lack of correction for multiple comparisons, although this is an explorative study with the aim of stimulating a change of perspective in PSP categorization. Future studies with larger sample size are needed to confirm our findings. Although we recruited patients in the Movement Disorder Unit with possible overestimation of motor symptoms, the extensive neuropsychological battery administered to the whole PSP group allowed us to identify the PSP-Cog phenotype, that can be overlooked during the clinical routine evaluation. Our decision to group patients with PSP-CBS, PSP-F, and PSP-SL under the term PSP-Cog is common to other studies and is based on the observation that the cortical subtypes and PSP-RS differ on tau brain distribution, while no dissimilarities were reported in CSF-tau or CSF-NFL (Jabbari et al. 2020; Guasp et al. 2021; Tsuboi et al. 2005; Kovacs et al. 2020).

In addition, PD-CFRS was developed for PD and has never been used in PSP. However, the impact of motor disability on ADL in PSP patients is even greater than in PD supporting the use this scale to independently assess motor and cognitive functional disability.

In conclusion, we found discrete clinical and imaging patterns that best characterize different PSP phenotypes defined according to established classification rules (Grimm et al. 2019). Within the time frame of our observation, we found worse clinical scores in PSP-Cog and PSP-PGF with the former presenting loss of autonomy, frequent occurrence of dementia and poor quality of life already after a few years of disease. Finally, we have further explored the presence and load of cognitive dysfunctions, assessed with an extensive neuropsychological battery, in expressing functional disabilities per sè and/or interacting with motor and clinical characteristics. We speculate that the identification of different clinical phenotypes may be expression of different progression patterns and in turn require tailored approaches in terms of follow-up and treatment. More research is also needed to identify discrete outcome measures including imaging to detect progression in each of the different phenotypes.

## Supplementary Information

Below is the link to the electronic supplementary material.

Supplementary file1 (DOCX 582 KB)

## Data Availability

All data are available upon reasonable request.
