# Supplementary material for: Clinical, cognitive, and morphometric profiles of progressive supranuclear palsy phenotypes
Source: J Neural Transm (Vienna). 2023 Jan 26;130(2):97–109. doi: 10.1007/s00702-023-02591-z (PMC9902314; doi:10.1007/s00702-023-02591-z)
Supplement: Supplementary file 1 — Supplementary file1 (DOCX 582 KB) [file 702_2023_2591_MOESM1_ESM.docx]

**e-Fig 1** Morphometric indices measurement and calculation


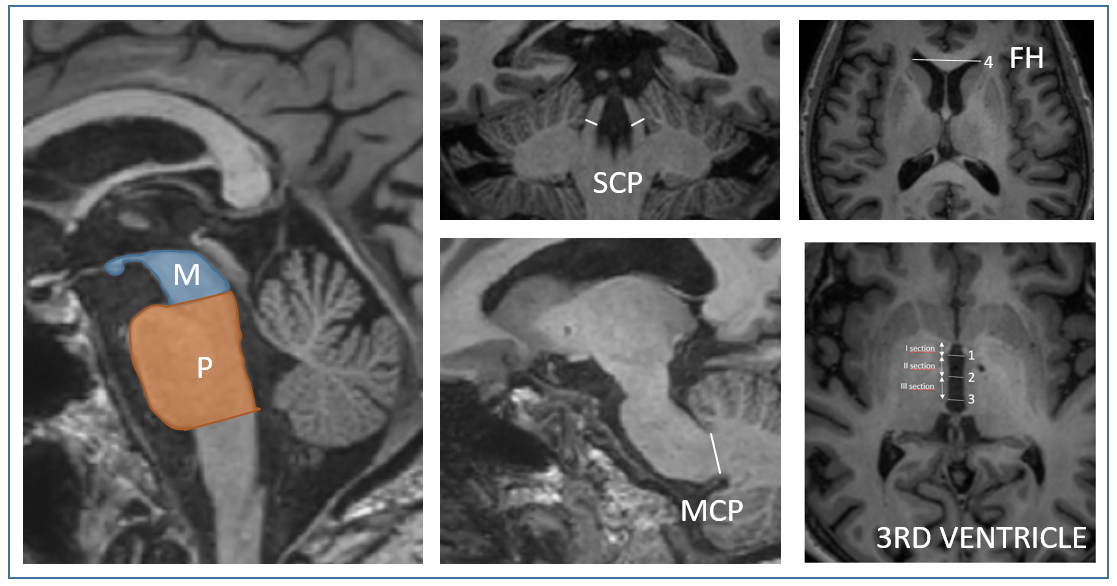


Note: Midbrain MRI assessments included mid-sagittal midbrain (M) and pons area (P), middle cerebellar peduncles (MCP) to superior cerebellar peduncles (SCP) ratio (MCP/SCP), the pons area (P) to midbrain area (M) ratio (P/M), the MR Parkinsonism Index (MRPI) = (P/M) × (MCP/SCP), the P/M 2.0 = (P/M) × (third ventricle width /frontal horns width (FH)), and the MRPI 2.0=MRPI × (third ventricle width /frontal horns width).

e-Table1. Normative ranges for MRPI and P/M (including 2.0 versions) morphometric indices (range 3-8 yrs.)

|  | **P/M ratio** | | **MRPI** | | **P/M 2.0** | | **MRPI 2.0** | |
| --- | --- | --- | --- | --- | --- | --- | --- | --- |
|  | Median | CI 95%* | Median | CI 95%* | Median | CI 95%* | Median | CI 95%* |
| **HC** | 3.08 | 2.45 - 3.72 | 7.67 | 4.99 - 10.55 | 0.56 | 0.26 - 0.85 | 1.42 | 0.68 -2.14 |
| **PSP-Cog** | 5.62 | 2.09 – 9.34 | 16.05 | 4.76 - 34.69 | 1.48 | 0.40 – 2.63 | 4.10 | 1.18 – 9.06 |
| **PSP-P** | 5.41 | 2.39 – 7.94 | 16.22 | 7.63 – 24.53 | 1.21 | 0.42 – 1.98 | 3.56 | 1.28 – 6.02 |
| **PSP-PGF** | 3.97 | 2.07 – 7.47 | 13.83 | 3.32 – 29.27 | 0.89 | 0.30 -1.98 | 2.75 | 0.40 – 7.34 |
| **PSP-RS** | 5.68 | 2.75 – 8,77 | 18.25 | 4.47 – 34.37 | 1.44 | 0.22 – 2.66 | 4.42 | 0.56 – 10.12 |

Note *: Confidence interval based on robust method (CLSI C28-A3) (bootstrap 10000 replication)

**e-Fig 2** Effect of scanner on morphometric measures among PSP phenotypes.


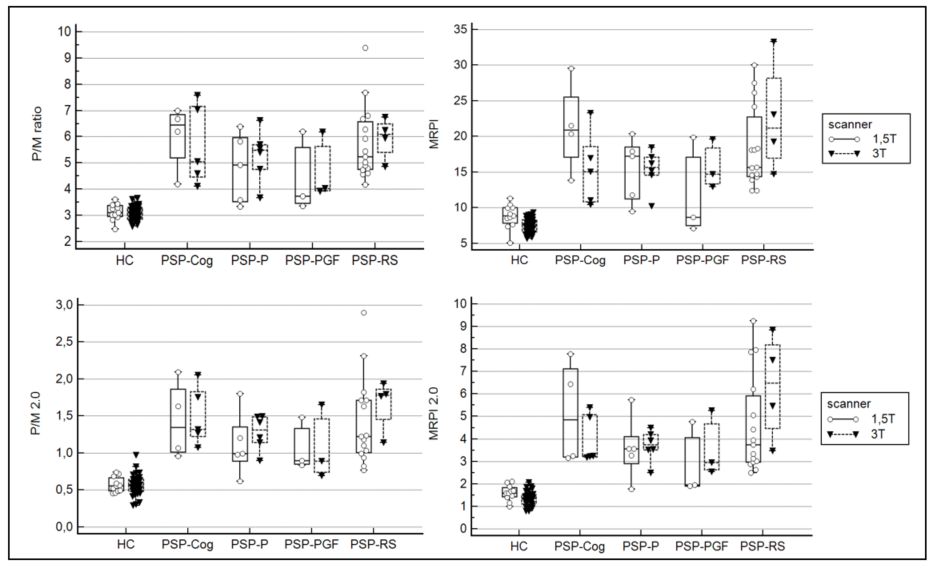


Note. Box plot with distribution of morphometric measures among PSP phenotypes by scanner at 5-year time point.

**e-Fig 3** Percentage of death across PSP phenotypes
